# Monitoring and Modeling Tree Bat (Genera: *Lasiurus*, *Lasionycteris*) Occurrence Using Acoustics on Structures off the Mid-Atlantic Coast—Implications for Offshore Wind Development

**DOI:** 10.3390/ani11113146

**Published:** 2021-11-04

**Authors:** Michael C. True, Richard J. Reynolds, W. Mark Ford

**Affiliations:** 1 Department of Fish and Wildlife Conservation, Virginia Polytechnic Institute and State University, Blacksburg, VA 24061, USA; 2 Virginia Department of Wildlife Resources, Verona, VA 24482, USA; rick.reynolds@dwr.virginia.gov; 3 U.S. Geological Survey, Virginia Cooperative Fish and Wildlife Research Unit, Blacksburg, VA 24061, USA; wmford@vt.edu

**Keywords:** tree bats, *Lasiurus*, *Lasionycteris*, wind turbine collisions, offshore, statistical modeling, monitoring, curtailment, prediction

## Abstract

**Simple Summary:**

“Tree bats” are North American bats that day-roost in trees year-round and undertake seasonal migration in lieu of hibernation. These bats have been shown to be highly susceptible to collisions with wind energy turbines and are known to fly offshore during migration. Therefore, as offshore wind energy expands off the eastern U.S. coast, there is some concern about potential impacts. We monitored bats in coastal Virginia, USA, using acoustic monitors—devices that collect the unique echolocation call signatures of bat species. We found that nightly tree bat visitation offshore or on barrier islands was associated with wind speed, temperature, visibility, and seasonality. Using statistical modeling, we developed a predictive tool to assess occurrence probabilities at varying levels of wind speed, temperature, and seasonality. Probability of occurrence and therefore assumed risk to collision is highest on high temperature and visibility nights, low wind speed nights, and during the spring and fall seasons. We suggest a similar modeling regime could be used to predict the occurrence of bats at offshore wind sites to inform potential mitigation efforts.

**Abstract:**

In eastern North America, “tree bats” (Genera: *Lasiurus* and *Lasionycteris*) are highly susceptible to collisions with wind energy turbines and are known to fly offshore during migration. This raises concern about ongoing expansion of offshore wind-energy development off the Atlantic Coast. Season, atmospheric conditions, and site-level characteristics such as local habitat (e.g., forest coverage) have been shown to influence wind turbine collision rates by bats onshore, and therefore may be related to risk offshore. Therefore, to assess the factors affecting coastal presence of bats, we continuously gathered tree bat occurrence data using stationary acoustic recorders on five structures (four lighthouses on barrier islands and one light tower offshore) off the coast of Virginia, USA, across all seasons, 2012–2019. We used generalized additive models to describe tree bat occurrence on a nightly basis. We found that sites either indicated maternity or migratory seasonal occurrence patterns associated with local roosting resources, i.e., presence of trees. Across all sites, nightly occurrence was negatively related to wind speed and positively related to temperature and visibility. Using predictive performance metrics, we concluded that our model was highly predictive for the Virginia coast. Our findings were consistent with other studies—tree bat occurrence probability and presumed mortality risk to offshore wind-energy collisions is highest on low wind speed nights, high temperature and visibility nights, and during spring and fall. The high predictive model performance we observed provides a basis for which managers, using a similar monitoring and modeling regime, could develop an effective curtailment-based mitigation strategy.

## 1. Introduction

Collisions with wind turbines are an expanding conservation concern for bats [1,2,3]. In North America, non-hibernating, migratory “tree bats” (Genera: *Lasiurus* and *Lasionycteris*) are particularly susceptible to collisions and are often the majority bat group in post-construction carcass surveys at wind energy facilities [4,5,6,7,8]. The tree bat mortality rate at wind turbines appears to be highly correlated with the seasonal movements of these species [9,10,11,12,13,14,15] whereby collisions are generally elevated in spring and maximized in fall migration periods [5,7,16]. Increased mortality counts during migration may be attributable to space-use increase due to fall mating and migration, erratic juvenile dispersal behavior, and general attraction to turbines [13,14,17,18].

North American tree bats are known to fly offshore with some regularity. This was first documented in anecdotal historical sightings from ocean vessels large distances off mainland coasts [19,20,21] and observations of tree bats on the island of Bermuda [22]. In the eastern North America, recent research has discovered high-flying tree bats 8.4–44 km from the main shoreline [23,24]. The occurrence of tree bats offshore and along shorelines follows a similar seasonal activity pattern to wind turbine collisions—a general peak during spring and fall migration [23,25,26,27,28]. The reasons for this behavior remains unknown but some speculate that the coastline serves as a topographic reference for navigation [29] or that favorable wind conditions over open ocean may aid in long distance migration [30]. It is posited that the eastern shoreline acts as a topographic barrier, concentrating southward migrating tree bats along the coast during fall [10].

Wind energy in the eastern United States is expanding at an accelerating rate, particularly in the offshore sector [31,32,33]. To date, two offshore wind turbine operations exist in the eastern United States that account for <50 MW capacity [33]. However, an increasing number of offshore projects are now leased and in the beginning construction phases. It is projected that these projects will account for more than 20 GW of rated capacity [33], a 400-fold increase. Although projections indicate offshore wind facilities will likely be concentrated in the wind resource rich Northeast, some development is proposed off the mid-Atlantic coast along Virginia, Delaware, Maryland, and New Jersey [33]. The impact this rapid development will have on bats is unknown, however, risk is certainly non-zero particularly for tree bats as they are the most susceptible bat group to collision (particularly during migration) and are the only bat group consistently seen at offshore localities (again, particularly during migration).

Onshore, extensive monitoring at wind facility sites post-construction have offered successful data driven conservation strategies to minimize bat mortality at turbines including, but not limited to, acoustic deterrents [34,35,36,37,38,39,40] and curtailment [41,42,43,44,45]. Curtailment is based on the knowledge that most bats generally avoid flying in overtly windy conditions, i.e., avoiding speeds generally above 5 m/s [44,45]. Therefore, at low wind speeds below this (or other) threshold(s), turbine managers feather turbine blades, bringing rotor movement to a minimum, and thereby minimizing bat fatalities. There has been some success in the use of curtailment to reduce bat mortality while also minimizing financial loss [42,44,46] through the development of “smart curtailment” algorithms. These are typically model based, multivariate approaches whereby curtailment is triggered by the expectation of high bat activity or probability of presence [42,47,48]. Although these strategies may hold promise for offshore wind energy impacts, unlike terrestrial systems, the factors that influence occurrence (and therefore the parameter values necessary to predict risk metrics) of bats offshore are poorly known.

Monitoring bat activity offshore is challenging. Passive acoustic monitoring over ocean waters requires some type of infrastructure (platforms, buoys, lighthouses, etc.) to support acoustic detectors. Barrier islands and offshore structures offer an alternative approach to collecting acoustic data in the near “offshore” environment if located a considerable distance from the mainland shoreline. Yet, these sites are accessible and feasible as detector deployment infrastructure. A few studies have approached the problem in this way, deploying acoustic detectors on islands, structures at sea, and on the coastline [26,27,28]. However, most of this research has been concentrated in the Northeast. Further south in the mid-Atlantic, some degree of seasonally fluctuating barrier island use by bats has been observed [25] as has fall offshore flight [23,24]. However, temporally, and geographically limited sample sizes somewhat constrain generalizability and the development of predictive models to describe the factors influencing bat use and to test the feasibility of a predictive curtailment algorithms in the near-offshore environment.

Our study sought to address these data gaps with a large sample of acoustic bat occurrence data off the Eastern Shore of Virginia (ESVA). From 2012 to 2019, the Virginia Department of Wildlife Resources deployed acoustic monitors at four barrier island sites and one offshore site. We used this large acoustic dataset to develop a model to describe migratory tree bat nightly occurrence relationships to season, atmospheric conditions, and site-specific characteristics. Our modeling served two purposes—description and prediction [49]. We describe the parameters that reveal the effects of various potential drivers of nightly occurrence of tree bats. Then, we use the model as a predictive tool of bat occurrence and hence potential risk for regional wind turbine collisions once deployed.

We hypothesized that tree bat occurrence in mid-Atlantic coastal environments is closely related to season due to the seasonal fluctuations in which tree bats use coastal landscapes and oceanic space. We predicted strong positive effects in spring and fall, moderate effects in summer, and negative effects in winter. We also developed competing hypotheses that the seasonal effect is explained by either (1) unique sites, or (2) the availability of local day-roosting habitat and potentially important foraging habitat (e.g., trees/forests, fresh water). We predicted that if the seasonal pattern is best explained by site specifics that unique sites would have noticeably different occurrence relationships to season. If the seasonal pattern is best explained by roosting habitat, sites with limited roosting habitat would have similar occurrence relationships to season (e.g., peaks only during migration). Lastly, we hypothesized that tree bat occurrence is closely related to multiple atmospheric conditions. We predicted that occurrence would be negatively related to wind speed, positively related to nightly temperature.

## 2. Materials and Methods

### 2.1. Study Area

**Figure 1:**
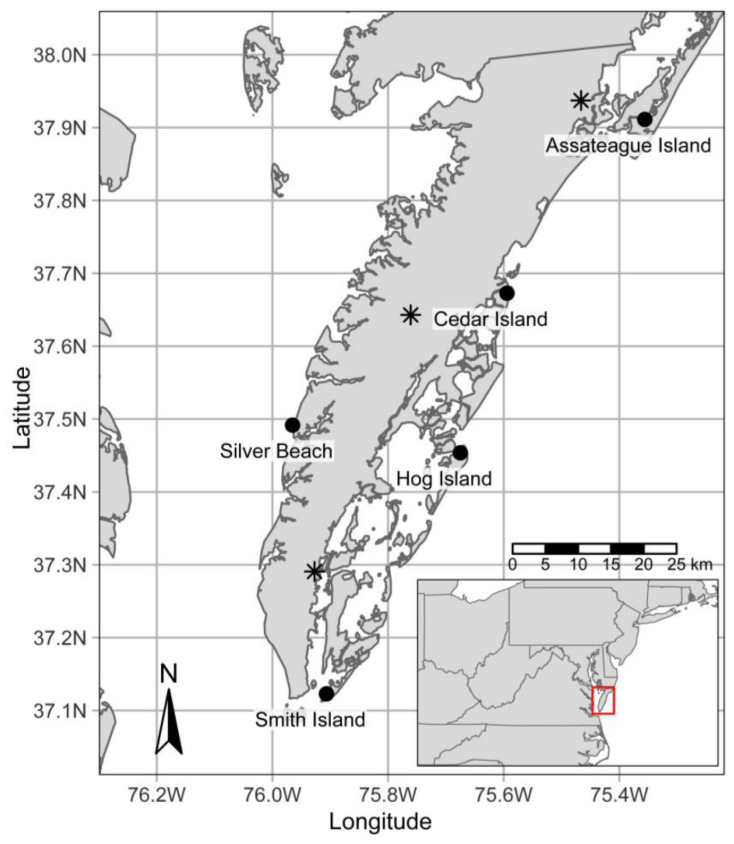
Acoustic detector sites (black points) on barrier islands and a light tower off the Eastern Shore of Virginia, USA, 2012–2019. Weather data was obtained from the nearest available National Oceanic and Atmospheric Administration (NOAA) weather stations (starred points) from the National Climatic Data Center (NCDC) online tool (Climate data online; https://www.ncdc.noaa.gov/cdo-web/; accessed on 9 November 2019).

**Figure 2:**
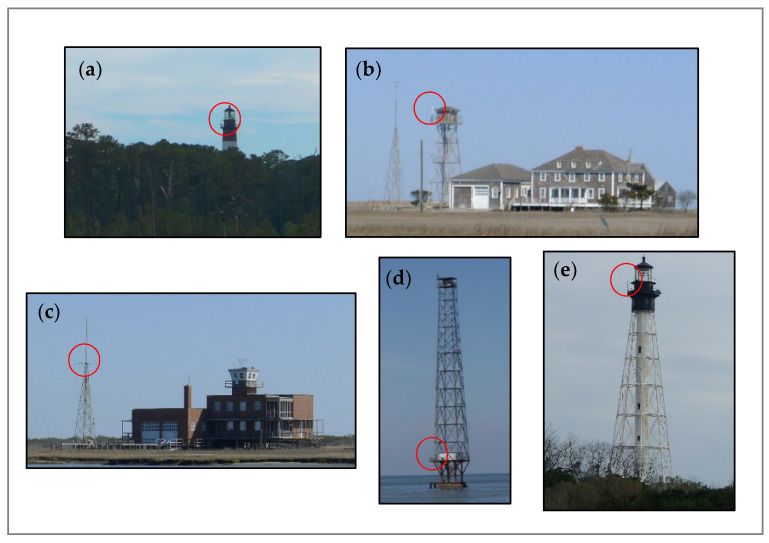
Lighthouses and structures serving as infrastructure for acoustic bat detector deployment on Eastern Shore of Virginia, USA, 2012–2019. Acoustic detector microphone locations are indicated by the red circles. Labels correspond to the locations given in Figure 1: (**a**) Assateague Island (Assateague Lighthouse); (**b**) Cedar Island (inactive United States Coast Guard station); (**c**) Hog Island (inactive United States Coast Guard station); (**d**) Silver Beach (offshore navigation light structure); (**e**) Smith Island (Cape Charles Lighthouse).

We conducted acoustic monitoring on four barrier island sites and one offshore site off the ESVA (Figure 1 and Figure 2) 2012–2019. The ESVA is the southern portion of the Delmarva Peninsula, surrounded by the Chesapeake Bay to the west and the Atlantic Ocean to the east. Locally, the vegetation is mid-Atlantic Coastal Plain deciduous and evergreen (pine) mixed upland and bottomland forest in its interior and intertidal saltmarsh habitat along the coasts. On the eastern Atlantic boundary, a chain of barrier islands occur that are characterized by little physical relief above sea level with upland shrub thickets, scattered patches of forest and salt marsh [50]. On the eastern side of ESVA, we monitored on Assateague Island on the Assateague Lighthouse, Cedar Island on an inactive United States Coast Guard (USCG) station, Hog Island on an inactive USCG station, and Smith Island on the Cape Charles Lighthouse. Cedar Island, Smith Island, and Hog Island are similar in that they are primarily composed of saltmarsh and upland shrub thickets. Some overstory evergreen vegetation exists on Hog Island, however, it is extremely limited in extent. In contrast to other ESVA study sites, Assateague Island has considerable deciduous and evergreen forest habitat. Additionally, Assateague Island contains fresh water sources. On the western boundary, we conducted research near Silver Beach on a navigation light structure approximately 0.7 km off the western shore of the ESVA in the Chesapeake Bay.

### 2.2. Acoustic Data

**Figure 3:**
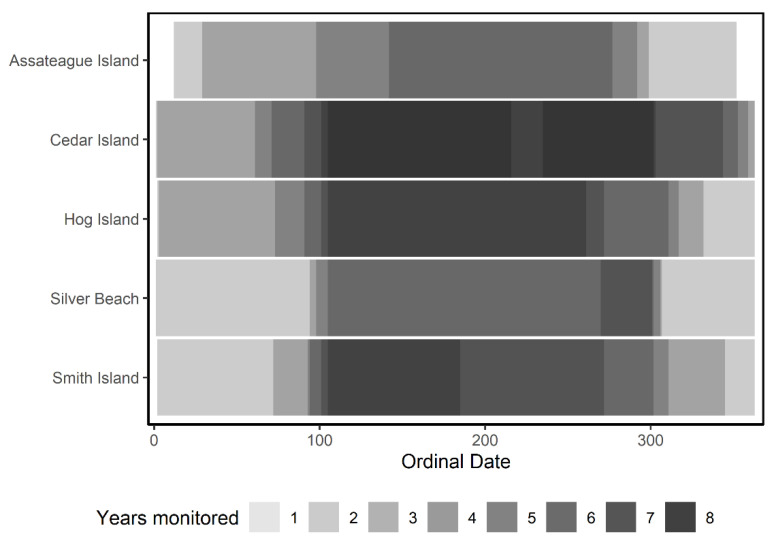
Site specific acoustic recording effort by ordinal date (day of the year). Effort (number of years monitored) is indicated as a heatmap of site vs. ordinal date such that lighter shades indicate lower effort (min = 1 year) and darker shades indicate higher effort (max = 8 years). Monitoring was conducted on the Eastern Shore of Virginia, USA, 2012–2019.

From 2012 to 2019, we collected acoustic data at the five ESVA sites named Assateague Island, Cedar Island, Hog Island, Smith Island, and Silver Beach (Figure 1 and Figure 2). We used frequency division/zero-crossing acoustic detectors (Anabat SD1 and SD2, Titley Scientific, New Ballina, NSW (any use of trade, firm, or product names is for descriptive purposes only and does not imply endorsement by the U.S. Government) that record high frequency (15–150 kHz) echolocation pulses of bats. We placed the detectors on existing structures (lighthouses or similar) at heights of approximately 10–40 m. We collected data annually typically across three seasons—beginning in early spring and through late fall and recorded during the winter season at least once per site (Figure 3). We considered acoustic recordings on a nightly basis from sunset to sunrise.

The post processing data structure was composed of timestamped individual echolocation sequences of bats (hereafter “bat passes” or “passes”). Bat passes is defined as a distinct series of echolocation pulses, or “clicks”, which is identified to one bat as they pass within range of the detector [51]. We used Kaleidoscope 4.5.0 Bats of North America—4.2.0 classifier (Wildlife Acoustic, Inc., Maynard, MA, USA) to identify passes to species, unidentified bat passes (“no ID”), or noise. We tallied nightly pass counts by individual species, no ID, and noise. To minimize false positives, we manually inspected subsets of passes identified to species to confirm identification. Due to the context of the problem and realization that >85% of identified passes were tree bats, we placed particular emphasis on correct identification of eastern red bats (*Lasiurus borealis*), silver-haired bats (*Lasionycteris noctivigans*), and hoary bats (*Lasiurus cinereus*), and only used tree bat pass data in our analysis. In wind turbine collision risk studies, recent evidence suggests that the hourly or nightly passage rates of bats pre-construction are poor predictors of fatality rates post-construction [52] so to account for this, instead of using hourly or nightly tallies of bat passes as our response variable, we restructured the data to consider only the binary occurrence (or non-occurrence) of tree bat(s) on a nightly basis.

### 2.3. Atmospheric Conditions and Other Variables

We compiled weather conditions from nearby National Oceanic and Atmospheric Administration (NOAA) weather stations on the ESVA (Climate data online; https://www.ncdc.noaa.gov/cdo-web/; accessed on 9 November 2019; Figure 1). We used the nearest available weather station to each site to approximate hourly weather conditions. We extracted hourly data on wind speed (m/s), wind gust speed (m/s), temperature (deg C), visibility (0–16.2 km), pressure (mmHg), precipitation duration (hours), precipitation (cm), and absolute humidity (mg/cm^3^). We filtered these data to reflect dates and hours in which the detector stations were active, i.e., reducing to a nightly basis between sunset and sunrise on active detector nights. We summarized each weather variable to reflect nightly conditions taking the nightly mean of wind speed, temperature, visibility, pressure, and relative humidity, the maximum wind gust speed, nightly cumulative sum of precipitation, and nightly cumulative number of hours precipitating. We also created a change in pressure variable calculated as the mean at the current night minus the mean of the previous night. Lastly, because bats may be more likely to be present at individual sites during different times of the year if they contain viable day-roosting habitat, we created a binary roosting habitat variable as has viable roost availability (forests) or, none or limited roost availability for each detector station. We also noted additional potentially relevant variables including ordinal date (day of year), site name, and year. We did not include the potentially relevant variable of detector height because we were limited by the number of unique heights (*n* = 5).

### 2.4. Presentation of Data and Exploratory Data Analysis

We performed an exploratory data analysis (EDA) to visualize the effect of wind speed, temperature, and seasonality. At each site, we noted tree bat occurrence or non-occurrence and calculated a 20-day moving average on the ordinal date (day of the year (1–366)). We also fit a smoothing line (generalized additive model (GAM) spline; [53]) to aid in visualization. We calculated 90%, 95%, and 99% quantile values in which 90%, etc. of all nights with tree bat occurrence were less than a wind speed threshold, greater than a temperature threshold, or between spring or fall date ranges. We calculated these quantiles for wind speed only, temperature only, and a mix of wind speed and temperature or a spring and fall date range.

### 2.5. Modeling

We used generalized additive models (GAMs; [54,55]) with a binomial distribution (logit link function) to model the relationship between binary nightly occurrence of tree bats and the variables. GAMs are an extension of generalized linear models (GLMs; [56]) such that the expected response is a link transformed summation of an intercept and the product of slope coefficients and variables, however, some or all variable effects may be specified as semi- or non-parametric real functions denoted as splines (hereafter “smooths” or throughout, “*f*(*x*)”; [54,55,57,58,59,60]). Smooths are created by a series of coefficient scaled basis functions “tied” together at knots—evenly spaced segments along the variable range. These smooths then, can take on complex, non-parametric shapes in the relationship between the variable effect and the variable as opposed to the generally linear, or parametric relationships in GLMs that may not reflect actual biological patterns. We performed all analysis in program R [61] and fit GAMs using the R package *mgcv* [53].

To test our first hypothesis and the subsequent competing hypotheses, we used a model selection process that tested three a priori models to select the appropriate model that best accounted for a potentially nonlinear seasonal effect. These three models contained smooth non-parametric function(s) of the ordinal date that took on factor level-specific shapes depending on the factor provided in the model. We supplied one model with no factor variable, one with roosting habitat availability that varied its intercept with site, and one with site only (Table 1).

We used the minimum change in Bayesian information criterion (∆BIC; [62]) as the basis for model selection because BIC outperforms Akaike’s information criterion (AIC) when *n* is large (*n* > 3000; [63]) and tends to select more parsimonious models because the penalty for complexity is larger than AIC (for *n* > *e*^2^). We calculated each a priori model BIC using Schwarz’s method of BIC = –2 *log*(*ℒ*) + *K log*(*n*) implemented in the *model.sel* function of the R package *MuMIn* [64].

We used the top a priori model structure in all further models as a baseline (i.e., this model structure was nested within any other further competing model). To include atmospheric conditions into the model, we first reduced variables by omitting those of high correlation (Pearson correlation coefficient > 0.7). We then created a global model that included all variables as smooth functions. We performed a dredge (i.e., fit all possible additive model combinations). We compared these models by BIC. We considered models <2 BIC points competing models in which we selected the top model by biological feasibility and interpretability [63].

To test predictive performance, we performed a series of diagnostic tests on the final model. First, to assess general performance, we conducted a Monte-Carlo cross validation (MCCV; [65]) on the area under the receiver operating characteristic (AUC; [66]). The AUC is a threshold dependent, sensitivity and specificity dictated metric of predictive performance for binary data such that an AUC of 0.5 is no better than random and an AUC of 1 is perfect prediction [66]. To perform the MCCV, we (1) randomly selected 85% of the data for training and 15% of the data for testing the model, (2) fit the model and predicted on the withheld data, and (3) measured and saved the AUC using the R package *pROC* [67,68]. We repeated those steps for 1000 iterations. We calculated the mean of the AUCs, and a 95% confidence interval by taking 2.5% and 97.5% quantiles of those 1000 iterations.

As a second metric of predictive performance, we again divided the data into 85% training and 15% testing groups. Using the training group, we fit the final model, then predicted occurrence probabilities from 0 to 1 on the testing group. We selected an optimal “cut-off” threshold using the Youdin index [69] to categorize occurrence. We used these categorizations to compare to their true occurrence values. Therein, we calculated a confusion matrix and values for sensitivity (true positive rate; true positives/true positives + false negatives) and specificity (true negative rate; true negatives/true negatives + false positives; [70,71,72]). We used various other R packages for data manipulation, cleaning, processing [73,74,75], visualizations, and mapping [76,77,78,79,80,81].

## 3. Results

We recorded acoustic data over eight years (2012–2019) resulting in a total of 5735 nights of recording across all detectors. We recorded at Silver Beach across four years (762 nights), Assateague Island and Smith Island across five years (791 and 1328 nights, respectively), Hog Island across six years (1268 nights), and Cedar Island across all eight years (1586 nights). Per year, effort was primarily centered on warmer months of spring to autumn, however, winter effort existed 1–2 years per site resulting in nearly entire year effort across all sites (Figure 3). We detected tree bats on a total of 39.26% of recorded nights, which varied by site (min = 29.89% at Hog Island, max = 71.30% at Assateague Island).

**Figure 4:**
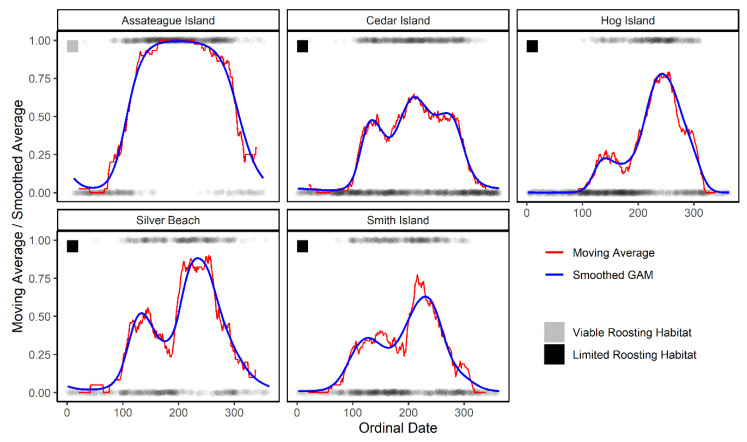
Raw occurrence data (black points: occurrence, non-occurrence (1, 0)) from acoustic detectors deployed on the Eastern Shore of Virginia, USA, 2012–2019. Data is grouped by site for all years of data collection stacked on a 1–366 ordinal date calendar. The raw data is shown as semi-transparent to visualize the occurrence density across years. Additionally overlaid are the results from the exploratory data analysis (EDA): the general relationship of tree bat nightly occurrence vs. ordinal date is represented by a 20-day two-sided moving average proportion of nightly occurrence (red line) and smoothed average using a generalized additive model (GAM) spline (blue line). Each site is labeled as containing viable day-roosting habitat (grey square) or limited day-roosting habitat (black square).

With respect to the exploratory data analysis, tree bats appeared to occur at sites with strong relationships to season (Figure 4). At Assateague Island, we detected nightly occurrence with a unimodal shape—low occurrence in winter, increase in spring, a peak in summer, and a decrease in fall. For all other localities, we detected bimodal effect shapes with respect to season—low occurrence in winter, a small peak in spring, a slight decrease in summer, then an increase and larger peak in fall. Sites showing a bimodal shape contained limited roosting habitat and probably limited foraging habitat.

The proportion of nights with tree bat occurrences appear related to wind speed and temperature as 90% of occurrences were on nights where wind speed averaged below 4.06 m/s and average temperatures were above 12.66 °C (Table 2). As an additive effect to these atmospheric conditions, the spring and fall months appeared to carry a large proportion of positive occurrence nights as 90% of occurrences occur during either wind speeds below 4.5 m/s, above 12 °C, or were between the dates of 28 April–14 May or 16 August–1 September.

Through model selection, the top approximating a priori model via minimum ∆BIC was model 2—the ordinal date shaped by roosting habitat model (Table 3). In post-hoc, our atmospheric variables reduced from seven to six potentially relevant variables by omitting nightly maximum wind gust (m/s) due to multicollinearity with nightly mean wind speed (m/s). We argue that nightly mean wind speed more closely relates to the wind conditions throughout an entire sampling night. We encountered missing values from weather stations that forced us to reduce the total number of nights from 5735 to 4864 so that each model used the same data in calculating model selection metrics.

Our global model included all remaining terms as additive smooths. The model selection dredge resulted in 128 models from which we selected the top model via BIC (Table 4). This top model included an intercept, site as a factor, a smooth effect of ordinal date based on day-roosting habitat, and smooth effects of nightly mean temperature, wind speed, and visibility.

**Figure 5:**
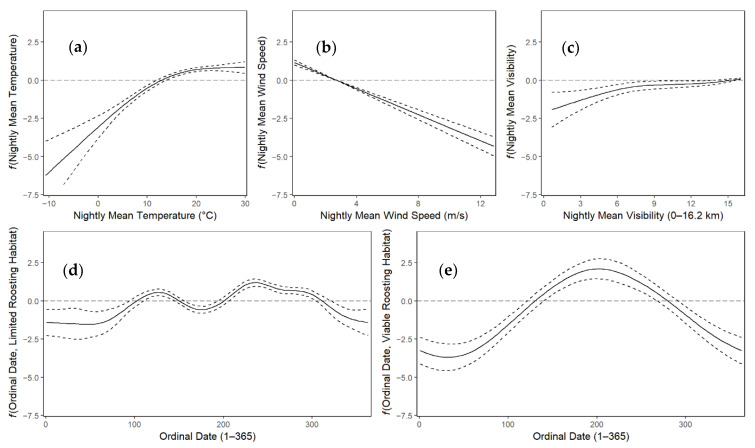
Visualizations of all smooth effect components, *f*(*x*), of the final model which was fit using acoustic data off the Eastern Shore of Virginia, USA, 2012–2019. The fits (solid lines) and 95% confidence intervals for the fits (black dotted lines) are displayed. Smooths, *f*(*x*), can be interpreted as the effect of the variable, *x*. Nightly occurrence probability is positively associated with larger smooth values along the range the of variable. These smooth components, *f*(*x*), are on the logit scale, for example, the dotted line at 0 on the logit scale indicates 50% probability of occupancy. The final model used smooth effects of nightly means of temperature (**a**), wind speed (**b**) visibility (**c**), and the ordinal date (**c**). The shape of the smooth effect was separated by roost availability, by limited (**d**), or viable (**e**).

The smooth effect of temperature and visibility was generally positive along the range of variable values, however, plateaus at higher values of each were evident (Figure 5). The smooth effect of wind speed was linear and negative along the range of variable values. The smooth effect of ordinal date was different for each roost availability type. For sites with limited roost availability the ordinal date effect was generally low in winter, locally maximized in spring at around ordinal date 125 (~May 5), lower in summer, and maximized in fall at around ordinal date 235 (~August 22). For sites with viable roosting habitat, the ordinal date effect generally increased from winter to spring, peaked in summer at around ordinal date 200 (~July 17), and decreased in fall (Figure 5). The intercept of the model was modified based on site. The greatest positive effect was Assateague Island (*β*_0_ + *β*_1_), the only site with viable roosting habitat (Table 5). The lowest effect was at Hog Island (*β*_0_ + *β*_2_; Table 5), the most distant barrier island from the ESVA mainland.

**Figure 6:**
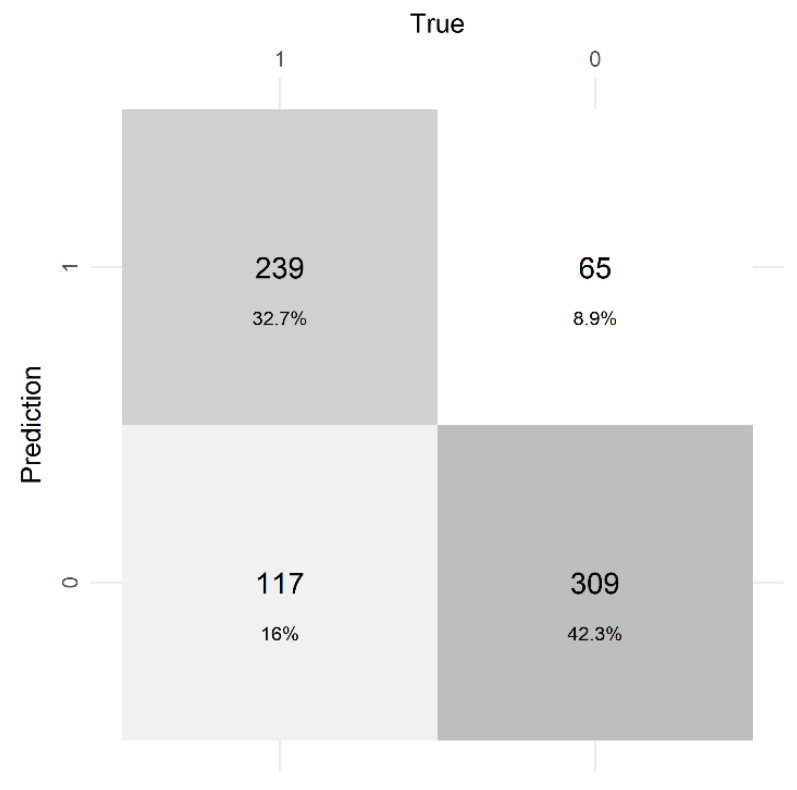
Confusion matrix of final model predictions compared to true values of test data collected off the Eastern Shore of Virginia, USA, 2012–2019. We used an 85% subset of the data to train the model (*n* = 4134) and a 15% subset to test the model (*n* = 730). Values and percentages are displayed as true positives (top left), false positives (top right), false negatives (bottom right), and true negatives (bottom left). Shading indicates more frequency of any categorization—darker indicating more weight.

Our final model was highly predictive. It contained a mean MCCV AUC value of 0.852–95% CI (0.828, 0.877). The optimal cutoff for predicting occurrence or non-occurrence was 0.393, which we used as a threshold to predict on withheld data. The model appeared to correctly predict occurrences as indicated by the confusion matrix (Figure 6). Therein, the number of false positives and false negatives were generally low (117 and 65 out of 730 data points). Sensitivity (true positive rate) and specificity (true negative rate) values were 0.826 and 0.671, respectively.

## 4. Discussion

Our hypotheses were generally supported by our analysis. First, tree bats do occur at offshore barrier island sites, but occurrence is most related to season. This became apparent as peaks in the occurrence rate over ordinal date contained local maximums in spring and fall. This seasonal effect is demonstrated in the EDA (Figure 4), the smoothed ordinal effects of the model (Figure 5), and in that 90 and 95% of occurrence nights fell within either nights of certain wind speed and temperature conditions or somewhat narrow spring or fall date ranges (Table 2). These seasonal effects undoubtedly are related to the migratory behavior of tree bat species [9,10,11,15,82,83]. Why tree bats traverse large bodies of water seasonally remains speculative, however, it could be explained by a simple increase in space use during migration or favorable conditions for long-distance flight occurring offshore [30]. Curiously, while both fall and spring seasons contain local peaks in occurrence, fall occurrence rates are higher than spring. This could be explained by the fact that fall is mating season and tree bats are more active in searching for mates and thereby more likely to explore more space [14,84]. This appears consistent as female eastern red bats are known to have multiple mates in a single season [85]. These effects are compounded, too, by additional volant juveniles navigating long distances for the first time. Moreover, these effects occur at a time when the species’ population should be at a level higher following summer parturition and juvenile volancy than winter and spring which could incidentally cause a higher rate of occurrence in fall as compared to spring [86].

Another obvious effect on occurrence was the presence or absence of viable roosting habitat (forests), which seemed to influence the shape of the seasonal pattern. The unimodal seasonal activity pattern observed at Assateague Island, which contained forests available for roosting habitat, was more typical of onshore sites—bats arrive in spring, activity peaks in mid-summer which corresponds to maternity activity, and bats settle into reduced activity states (cave hibernation (cave bats) or intermittent torpor (tree bats)) in fall and then winter [11]. The other survey sites that contained little or no forest patches seemed to be visited consistently in just spring and fall—an indication of vagrant, rather than maternity, use. These sites contained lower activity in general, suggesting that without quality roosting habitat, bat occurrence and residency time was low, aside from the spring and fall season. Therefore, our results support the latter of our competing hypothesis—the pattern of seasonal use is best explained by the availability of local day-roosting habitat. This point also supports that siting for offshore wind turbines should consider increasing distance to viable roosting habitat to reduce curtailment needs during the summer. A similar study also observed this [27], that bat activity decreases with increasing distance from mainland and decreasing forest coverage.

Next, including nightly atmospheric conditions greatly improved the model. It was not surprising that wind speed had negative effects on occurrence and conversely temperature and visibility had positive effects on occurrence. For example, we found that ~95% of nights that contained positive tree bat occurrence were <~5 m/s (~11 mi/hr) and >~10 °C (50 °F). High wind speeds and low temperatures greatly increase the energy costs associated with flying [87] which may be particularly true at distant barrier islands where we speculate that the nightly origin of these bats was most likely non-local, i.e., from the ESVA mainland. We understand that a multitude of atmospheric conditions relate to the activity states of bats [28] and the migratory behavior of birds [30]. Indeed, many observations of over-ocean flying bats have been during calm conditions [88]. We were initially surprised that visibility was selected as a relevant variable considering that bats rely on audible cues to navigate during flight via echolocation. However, it is intuitive to assume that bats use visual cues when flying above the ocean and/or when traveling to the islands and structures that we detected them nearby. Bats are known to echolocate while traveling over the ocean, particularly when close enough to detect them with acoustics (e.g., [23,89]), however, hoary bats *(Lasiurus cinereus*) sometimes forgo echolocation when traveling, and therefore rely solely on visual clues intermittently [90]. It is not beyond the realm of possibility that over-ocean flying bats use vision when there are no reflective surfaces for echolocation (e.g., at high altitudes) and therefore are unlikely to engage in over-ocean flights when visibility is low. The negative relationship of occurrence to visibility could also be explained by poor conditions for flying in general (rain, wind, low temperatures) as poor visibility is generally associated with those poor weather conditions, which, require more energy to fly in (e.g., rain, [91]).

Our modeling effort increased our understanding of the pattern of occurrence of migratory tree bats at barrier island sites in the mid-Atlantic. Importantly, this dataset revealed the conditions whereby occurrence along the coast is more or less likely. Whether inland or coastal, it is established that site characteristics, seasonality, and atmospheric conditions influence the activity rates of bats [25,26,27,28]. These effects are reinforced with our findings at the more southerly latitude of the ESVA. The occurrence of bats offshore was highly predictable when using the model. Our large AUC values from the MCCV indicated that, on average, given site specifics, day of the year, and atmospheric conditions, the occurrence probability of migratory tree bats is very accurate for the ESVA sites. We also argue that our study continues a trend of consistency across studies. Tree bats appear to use offshore areas on the east coast during a certain set of conditions—calm and warm weather, during fall (and to some extent spring), and nearer to shorelines or forest coverage than far [27,88]. Therefore, we believe our results are fairly generalizable to the surrounding region of the mid-Atlantic coastline.

Nonetheless, our study is not without limitations. First, observing bats via acoustics contain potential biases in that the physics of ultrasonic sound (bat echolocation pulses) change with atmospheric conditions [92]. This, plus the fact that non-occurrence does not necessarily equate to absence (i.e., detection probability is not reliably 1; [93]), may over or underestimate probabilities of occurrence depending on the conditions, time of year, among other factors. Additionally, acoustic activity of bats and wind turbine collision risk are not always analogous [52]. Regardless, these issues largely concern the correct detection of absence rather than presence of bats. In our research, nights of known occurrence follow patterns that are consistent (i.e., prediction accuracy is high on withheld data when trained on multiple years of data). Lastly, our study was limited in the number of sites to support our results. As we were limited in detector deployment infrastructure and accessibility, we were restricted to only five sites which could restrict generalizability and could contain bias. As just mentioned however, our study does not differ in major ways from other studies. Even with just five sites, patterns of occurrence follow associations with atmospheric conditions, site specifics, and seasonality in a largely nonunique manner which, as a standalone study may suffer with site limitations, but in the greater literature is in support of what has previously been known [25,27,89].

The development and deployment of predictive smart curtailment algorithms is currently underway onshore and may be a viable method to reducing bat collisions at offshore wind farms. While additional research is warranted to assess collision risk at project-level localities, these data and this analysis helps identify a starting-point in assessing the temporal and climatic conditions when tree bats may be most susceptible to impacts from wind turbines offshore in the Mid-Atlantic region. If nightly occurrence does indeed generally correlate to offshore wind strike risk, a similar algorithm or model could be used as a to predict when risk is more likely. Even more simply, if managers were to implement simple standards, such as curtailing on nights with average wind speeds <5 m/s, temperatures >10 °C, and/or during the spring and (especially) the fall, most bat occurrence (and potential risk) could be avoided. It appears that curtailment using a combination of variables as these could be a relatively inexpensive [94] and effective [44,95] way to reduce bat fatalities at offshore wind facilities.

## 5. Conclusions

Although we do not suggest using our specific model as a smart curtailment tool per se, this framework provides a viable starting point for creating curtailment regimens in the Mid-Atlantic. Our model was highly predictive and parsimonious which may suggest generalizability. Our results suggest that tree bat occurrence, and therefore a potential for risk is most likely under general and definable conditions—during the spring and fall seasons and on nights with low wind speeds, high temperatures, and high visibility. As such, it would be feasible for wind energy managers to collect acoustic data pre- and post-construction, assess the frequency of visitation at their specific sites, use site specific effects, atmospheric conditions, and seasonality in a modeling framework, and test the predictive ability of the model for specific locations. Using this approach, managers could have some basis for understanding which conditions influence nightly occurrence and when and where bat collision risk is non-zero or high as a guide to curtailment or other mitigation practices to minimize bat mortality.

## Figures and Tables

**Table 1:** A priori generalized additive models to evaluate the shape of the effect of seasonality (ordinal date) of tree bat occurrence at offshore/barrier island sites, Eastern Shore of Virginia, USA, 2012–2019. Three models are presented—one that only considers the ordinal date, one that considers local roosting availability, and one that considers site specifics.

Model Name (Model Number)	Explanation	Biological Significance
Ordinal date only (1)	Nightly tree bat occurrence is explained by a smoothed effect of the ordinal date.	Tree bat visitation of barrier islands is related to the day of the year (ordinal date) because of the seasonal offshore habits of tree bats during migration.
Ordinal date by roosting habitat (2)	Nightly tree bat occurrence is explained by a smoothed effect of the ordinal date, but two shapes exist—one for viable roosting habitat, one for limited roosting habitat. Additionally, an intercept modifier exists for each site.	Tree bat visitation of barrier islands is related to the day of the year (ordinal date) because of the seasonal offshore habits of tree bats during migration. For sites with viable roosting habitat, the effect of ordinal date is likely highest in mid-summer, indicating maternity use. For sites with limited roosting habitat the effect of ordinal date is likely highest in spring and fall, indicating migratory use only. While the effect shapes are roost-availability specific, occurrence rates may differ between sites for some unknown reason, so the intercept is free to fluctuate between sites.
Ordinal date by site (3)	Nightly tree bat occurrence is explained by smooth effects of the ordinal date—one for each site.	Tree bat visitation of barrier islands is related to the day of the year (ordinal date) and this relationship is specific to each site. For instance, some sites may be migration only, some sites may be for some migration and summer use, and many other minute differences between sites.

**Table 2:** The 90%, 95%, and 99% quantiles representing the proportion of nights with tree bat occurrence under certain conditions of wind speed, temperature, and/ or date ranges using acoustic data collected on the Eastern Shore of Virginia, USA, 2012–2019.

Percent of Positive Occurrence Nights with Conditions	Conditions	Values
0.90	Wind Speed	<4.06 m/s
0.95	Wind Speed	<4.90 m/s
0.99	Wind Speed	<7.28 m/s
0.90	Temperature	>12.66 °C
0.95	Temperature	>10.31 °C
0.99	Temperature	>5.43 °C
0.90	Wind Speed and Temperature or Date Range	<4.5 m/s and >12 °C or within either 28 April–14 May or 16 August–1 September
0.95	Wind Speed and Temperature or Date Range	<4.5 m/s and >12 °C or within either 23 March–11 June or 11 July–7 October
0.99	Wind Speed and Temperature or Date Range	<4.5 m/s and >12 °C or within 24 February–3 November

**Table 3:** A priori models ranked by Bayesian information criterion (BIC) from acoustic data collected off the Eastern Shore of Virginia, USA, 2012–2019. Displayed are the model names and numbers as referenced in Table 1, approximate degrees of freedom, −*log* (Likelihood), BIC, and ∆BIC.

Model Name (Model Number)	df	*log* (ℒ)	BIC	∆BIC
Ordinal date by roosting habitat (2)	18	−2798.92	5758.08	0.00
Ordinal date only (1)	14	−2834.04	5794.93	36.85
Ordinal date by site (3)	44	−2760.76	5906.33	148.25

**Table 4:** Top five competing post-hoc models ranked by Bayesian information criterion (BIC) from acoustic data collected off the Eastern Shore of Virginia, USA, 2012–2019. The model selection dredge contained all possible combinations of site (factor, 5 levels), smooth effects, *f*(*x*), of ordinal date (1–365; one for each roost availability type [viable, limited]), and smooth effects of nightly mean pressure (mmHg), temperature (C), visibility (0–10 mi), and wind speed (m/s), total precipitation duration (hours), and change in pressure from the previous night. N/I indicates no inclusion in that particular model. Terms with “+” indicate inclusion of that term in the model as a smooth function. We displayed the model degrees of freedom (df), BIC, and ∆BIC.

Intercept	Site	*f* (Ordinal, by Roost Habitat)	*f* (Press)	*f* (Temp)	*f* (Visib)	*f* (Wind Spd)	*f* (Precip Duration)	*f* (∆Press)	df	BIC	∆BIC
−1.149	+	+	N/I	+	+	+	N/I	N/I	23	4557.7	0.00
−1.149	+	+	N/I	+	+	+	+	N/I	24	4563.8	6.09
−1.136	+	+	N/I	+	N/I	+	N/I	N/I	20	4567.5	9.82
−1.134	+	+	N/I	+	N/I	+	+	N/I	21	4569.1	11.37
−1.149	+	+	+	+	+	+	N/I	N/I	28	4579.8	22.12

**Table 5:** Beta parameters names, estimates, standard errors, and *p*-values for site-specific intercept modifiers in the final model fit using acoustic data off the Eastern Shore of Virginia, USA, 2012–2019. Estimates of *β*_1_–*β*_4_ should be added to the intercept (*β*_0_) to interpret the intercept modifying effect of the specific locality correctly.

*β* Parameter	Estimate	Standard Error	Z-Score	*p*-Value
*β*_0_ (Intercept, Cedar Island)	−1.170	0.086	−13.69	<0.05
*β*_1_ (Assateague Island)	3.133	0.209	14.96	<0.05
*β*_2_ (Hog Island)	−0.444	0.101	−4.38	<0.05
*β*_3_ (Silver Beach)	0.311	0.113	2.74	<0.05
*β*_4_ (Smith Island)	−0.335	0.104	−3.21	<0.05

## Data Availability

Cleaned data (nightly values of occurrence or non-occurrence and their associated predictor variables) and supporting R scripts to run all analyses are provided at a github repository (github.com/mtrue/vacoastalbats; accessed on 29 September 2021). Raw acoustic data is available upon request. E-mail the corresponding author for details.
